# Structural Model of the Oncostatin M (OSM)-OSMRβ-gp130 Ternary Complex Reveals Pathways of Allosteric Communication in OSM Signaling

**DOI:** 10.2174/0109298673362742250508113436

**Published:** 2025-07-01

**Authors:** Qingqing Du, Ding Luo, Weiwei Xue, Yan Qian

**Affiliations:** 1 Department of Pharmacy, The Second Affiliated Hospital of Chongqing Medical University, Chongqing, 400010, China;; 2 Chongqing Key Laboratory of Natural Product Synthesis and Drug Research, School of Pharmaceutical Sciences, Chongqing University, Chongqing, 401331, China

**Keywords:** Oncostatin M signaling, protein structure prediction, molecular dynamics simulation, allosteric communication network, drug design, heterodimer receptor

## Abstract

**Introduction:**

Human oncostatin M (OSM) is a pleiotropic cytokine that regulates inflammatory and immune responses by binding to the heterodimer receptor complex OSM receptor beta (OSMRβ) and glycoprotein 130 (gp130). The distinct signaling pathways triggered by OSM are involved in multiple chronic inflammatory conditions, such as inflammatory bowel disease (IBD), rheumatoid arthritis (RA), and cancers, making the OSM-bound receptor complex a significant therapeutic target. Currently, no 3D structure of human OSM recognition complex is available, and thus, the molecular mechanisms underlying OSM signaling remain poorly understood.

**Methods:**

In this study, for the first time, we proposed a full-length structural model of the human OSM-OSMRβ-gp130, generated using AlphaFold2 protein structure prediction and all-atom molecular dynamics (MD) simulation (~ 1.12 million atoms with explicit solvent), enabling investigation of the geometric and dynamic profiles of OSM-OSMRβ-gp130 structure at atomic-level.

**Results:**

Analysis of the simulation trajectory demonstrated that the structural rearrangements of the heterodimer receptors (*i.e.*, OSMRβ and gp130) initiated by OSM binding mediated the signal transduction from the extracellular to the intracellular domains. In the representative conformation identified through clustering analysis, two main allosteric pathways contributed were found to mediate signal transduction from the allosteric region of OSM to the active sites of OSMRβ and gp130. Finally, two druggable binding sites located on OSM and gp130 were detected by dynamically monitoring pocket flexibility throughout the simulation. A comprehensive analysis of the OSM-OSMRβ-gp130 model was carried out with respect to OSM signaling.

**Conclusion:**

The findings of this study not only enhance the mechanistic understanding of OSM binding to the heteromeric OSMRβ/gp130 but also identify druggable binding sites for structure-based design of small molecules to inhibit the intracellular signal transduction.

## 1 INTRODUCTION

**Fig. (1):**
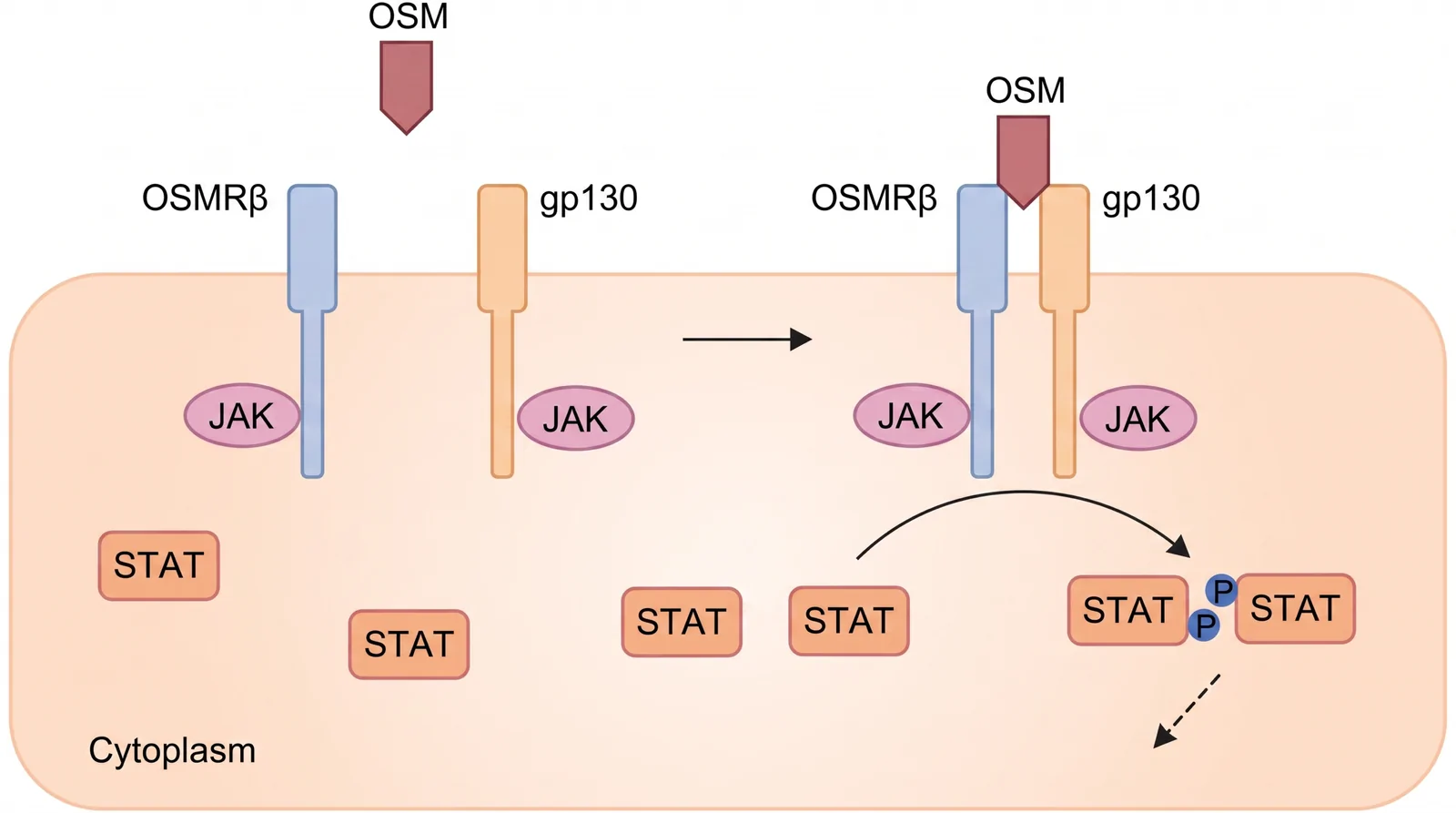
OSM binding and signaling pathways. OSM specifically binds with high affinity to the heterodimeric receptor complex composed of OSMRβ and gp130 and subsequently activates signaling pathways, such as Janus kinase (JAK)/signal transducer and activator of transcription (STAT).

Oncostatin M (OSM) is a pleiotropic cytokine, exhibiting both pro- and anti-inflammatory effects, and is a member of the interleukin 6 (IL-6) cytokines family [1]. There are 9 members in the IL-6 family, including IL-6, IL-11, OSM, ciliary neurotrophic factor (CNTF), leukemia inhibitory factor (LIF), cardiotrophin 1 (CT-1), cardiotrophin-like cytokine (CLC), IL-27, and IL-31 [2]. Notably, cytokines of the IL-6 family share the common signaling receptor subunit glycoprotein 130 kDa (gp130) [3]. As a representative member of the IL-6 family in humans, OSM only binds to the heterodimer receptors (complex of OSMRβ and gp130) with high affinity [4] and triggers the activation of the signaling pathways of Janus kinase (JAK)/signal transducer and activator of transcription (STAT) (Fig. **1**) [5]. Thus, OSM signaling has been regarded as a promising therapeutic target in multiple chronic inflammatory conditions, such as inflammatory bowel disease (IBD) [6], rheumatoid arthritis (RA) [5], and cancers [7].

In recent years, several monoclonal antibodies (mAbs), for example, GSK315234, GSK2330811, and vixarelimab targeting OSM or OSMRβ, have been developed and entered into clinical trials [5]. Although those neutralizing mAbs provide novel insights into the therapies for chronic inflammatory diseases [3], the administration of current biologics (*e.g.*, intravenous and subcutaneous) is difficult and has several limitations in terms of patient-friendliness, convenience, and cost-effectiveness [6]. More recently, the discovery of novel modulators that regulate extracellular cellular signal response induced by protein-protein interactions (PPIs) has attracted great attention from academia [8-11]. For example, the idea of rational design of orally bioavailable small molecules [12] or miniproteins [13] targeting cytokines and receptors shows potential to tackle the problems in treatment [14, 15]. However, for rational (structure-based) drug design, the identification of druggable sites on OSM and receptors [16-19] relies heavily on the conformational ensembles of these PPIs [20]. Currently, the structural basis of the OSMRβ-gp130 and OSM-OSMRβ-gp130 remains unresolved [4, 21], resulting in the mechanism by which OSM activates downstream signaling through allosteric communication poorly understood, which hinders the structure-based design of novel modulators targeting OSM signaling [22]. Therefore, it is urgently needed to investigate the protein structures and conformational changes underlying OSM signaling at the atomic level.

**Fig. (2):**
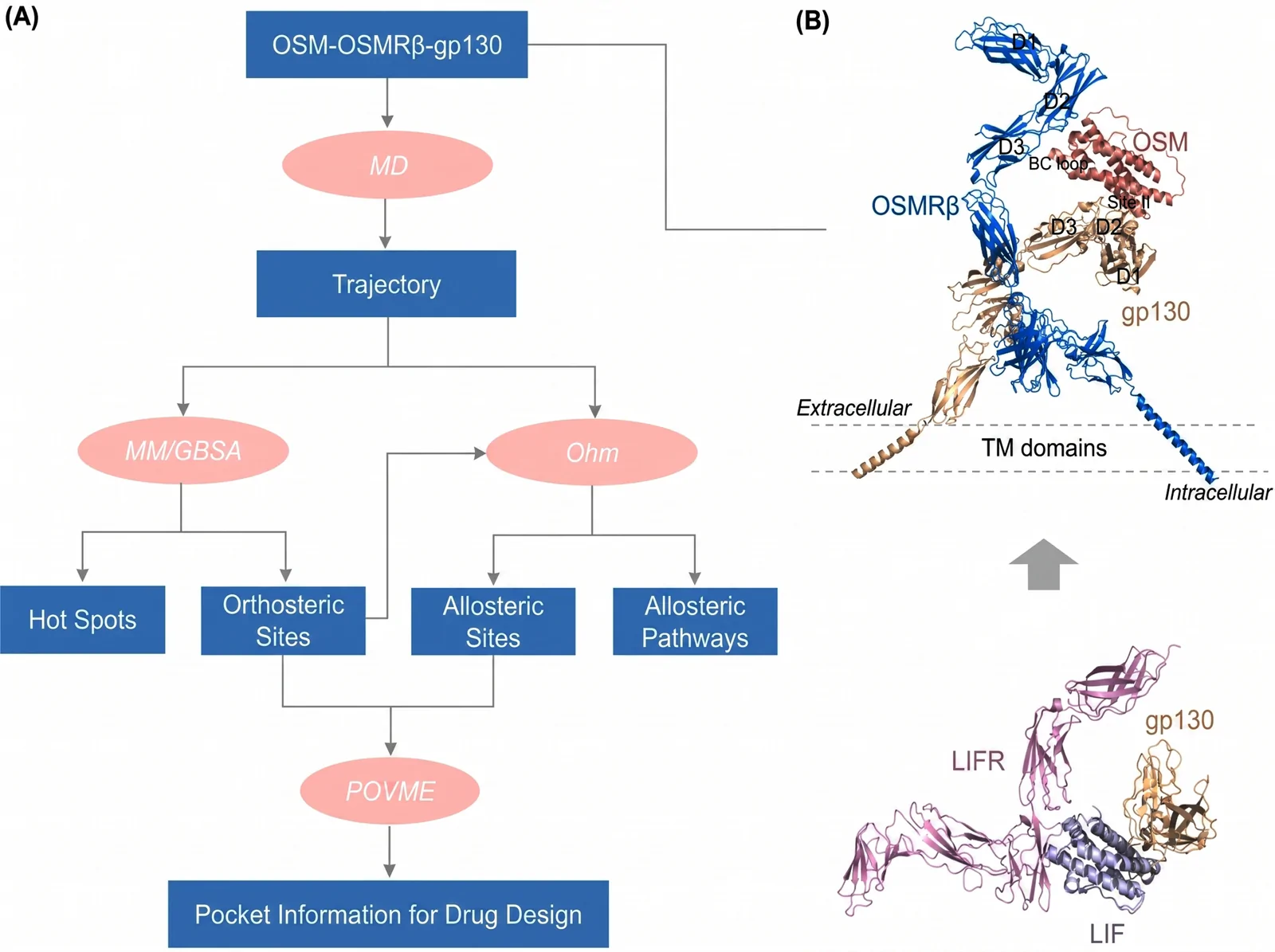
Computational modeling of an OSM-OSMRβ-gp130 complex structure. (**A**) A computational workflow used in this work for investigating the structural dynamics and allostery of OSM-OSMRβ-gp130. (**B**) Construction of the OSM-OSMRβ-gp130 model by using the crystal structures of LIF-LIFR (PDB ID: 2Q7N) and LIF-gp130 (PDB ID: 1PVH) as templates.

In this study, we proposed a structural model of OSM-OSMRβ-gp130 by the state-of-the-art protein structure prediction algorithm AlphaFold2 [23] and all-atom molecular dynamics (MD) simulation (Fig. **2A**). First, using the experimental homologues structures of LIF in complex with LIFR [24] and gp130 [25] as templates, the ternary model was constructed through manual docking of OSM onto the OSMRβ and gp130. Then, the initial structures of the complex immersed in an explicit solvent environment (~ 1.12 million atoms) were carefully sampled by all-atom MD simulation. Finally, an advanced analysis of the simulation trajectory was conducted, yielding intriguing results. These include: (i) large-scale conformational changes occurred on the OSMRβ, (ii) hot spots that are important for OSM and OSMRβ, as well as OSM and gp130 recognitions, (iii) allosteric pathways, which OSM binding propagate *via* signal transduction from the OSM to OSMRβ and gp130, and (iv) potential druggable sites on OSM and gp130. Overall, the proposed structural models provide key insights into the molecular architectures of protein-protein recognition underlying the OSM signaling and reveal promising binding pockets for the rational design of drugs with novel mechanisms of action.

## 2 MATERIALS AND METHODS

### 2.1 Systems Setup

#### 2.1.1 Construction of the OSM-OSMRβ-gp130 Model

The 3D structures of full-length OSM (AF-P13725-F1-model_v3), OSMRβ (AF-Q99650-F1-model_v3), and gp130 (AF-P40189-F1-model_v3) were obtained from AlphaFold Protein Structure Database [26]. The residues in OSM (1-25, 213-252), OSMRβ (661-918), and gp130 (779-979) with the predicted local-distance difference test (pLDDT) score less than 70, which were not involved in PPIs, were discarded [27].

To construct the initial models of OSM-OSMRβ-gp130, the crystal complex structures of LIF-LIFR (PDB ID: 2Q7N) and LIF-gp130 (PDB ID: 1PVH) [24] were retrieved from RCSB PDB database [28] and used as templates. First, the structure of LIF-LIFR was aligned with PyMOL [29] to obtain the ternary complex of LIF-LIFR-gp130 using LIF as a reference. Then, the structures of the truncated OSM, OSMRβ, and gp130 were aligned with LIF, LIFR, and gp130 in the ternary complex, respectively. Finally, the ternary structure model of OSM-OSMRβ-gp130 was built by saving the corresponding proteins as independent PDB files.

#### 2.1.2 Preparation of the Lipid Bilayer Environment and Simulation Box

The constructed model structure of OSM-OSMRβ-gp130 was first pre-oriented in OPM [30], which was designed to provide detailed spatial orientations of membrane proteins relative to the lipid bilayer's hydrocarbon core. Then, the complex structure was inserted into an explicit POPC lipid bilayer with 1338 lipids using CHARMM-GUI Membrane Builder [31]. The TIP3P water of 40 Å thickness was placed above and below the constructed bilayer, and the Na^+^ and Cl^−^ counterions were used to neutralize the systems at an environmental salt concentration of 0.15 M, resulting in the whole system forming a periodic cell (215 × 215 × 254 Å3) containing 1,116,528 atoms for OSM-OSMRβ-gp130 system. The characterization of protein, POPC, and water was carried out using AMBER ff14SB [32], Lipid17 [33], and the TIP3P water model [32].

### 2.2 Molecular Dynamics Simulations

All MD simulations were carried out using a GPU-accelerated version of the AMBER22 package. The input files for the AMBER simulation were generated in CHARMM-GUI [34]. For each system, the whole simulation contained three main steps: minimization, equilibration, and production run. First, the initial structure was minimized for 5,000 steps with positional restraints of 10 kcal mol^−1^ Å^−2^ applied to the protein backbone atoms. Second, the equilibration was performed for 15 ns through 6 continuous short runs (2.5 ns). During equilibration, the force constant used for positional restraints of the protein backbone atoms was linearly reduced from 10 kcal mol^−1^ Å^−2^ to zero. Third, a 200 ns production simulation was performed in the NPT ensemble at 310 K and 1 atm, using periodic boundary conditions. The temperature and pressure were controlled with a Langevin thermostat and a Monte Carlo barostat, respectively. Electrostatic interactions with a distance cutoff of 9 Å were calculated using the Particle-Mesh Ewald (PME) method [35]. The SHAKE algorithm [36] was used to keep all-bonds constraints, and the time step was set as 2.0 fs.

### 2.3 Trajectory Analysis

Snapshots extracted at regular intervals from each MD simulation were used for root-mean-square deviation (RMSD), RMSF, and k-means clustering analysis.

#### 2.3.1 RMSD Calculation

The RMSDs of ligand (heavy atom) and protein (Cα) as a function time were calculated with CPPTRAJ [37] on 1000 snapshots extracted from the 100,000 frames during each simulation. Before RMSD calculation, each of the 1000 snapshots was aligned to the reference (representative) structure obtained from the cluster analysis.

#### 2.3.2 K-Means Clustering Analysis

For each trajectory, the clustering analysis was performed with CPPTRAJ [37] on 10, 000 snapshots extracted from the 100,000 frames using the unsupervised k-means algorithm. The parameters of “randompoint maxit” and “sieving” were set to 500 and 10, respectively. The RMSD of the ligand’s heavy atoms was used as distance metric. The clustering was finished when the number of clusters reached 10.

### 2.4 Identification of Hotspots Residues

The energy between ligand and protein of the 1,000 snapshots during the simulation was estimated with mm_pbsa.pl using MM/GBSA method [38, 39], as mentioned below (1):

Δ*G*MM/GBSA = Δ*E*ele + Δ*E*vdW + Δ*G*pol + Δ*G*nonpol (1)

Where ΔEvdW and ΔEele are the van der Waals and electrostatic interaction energies, and Δ*G*pol and Δ*G*nonpol are the polar and non-polar solvent energies, respectively. Δ*E*vdW and Δ*E*ele were calculated using the AMBER ff14SB [32] in the gas phase. Δ*G*pol was calculated by solving the GB equation with the dielectric constants of solute and solvent set to 1 and 80 [40], respectively. Δ*G*nonpol was calculated by using Δ*G*nonpol = γ × Δ*SASA*, where γ = 0.005, and *SASA* is referred to the solvent-accessible area and determined using a water probe radius of 1.4 Å [40].

### 2.5 Allostery Calculation

Based on the top representative structure of the OSM-OSMRβ-gp130 complex from K-means clustering, Ohm’s method [41] was used to perform allostery analysis, including the prediction of allosteric sites, identification of allosteric pathways (communication networks), and determination of critical residues in the allosteric pathways. First, Ohm’s algorithm extracted all the atom-wise contacts (two atoms within 3.4 Å are counted as a contact). Then, by defining the active site information, a contact matrix and a perturbation propagation probability matrix were calculated to obtain the frequency of the allosteric coupling intensity (ACI). The allosteric site can be identified through residues (nodes) with high ACI values. Finally, by providing information on the residues of both the orthosteric site and the allosteric site, the allosteric pathways and the critical residues connecting the active site and the allosteric site were determined using Ohm’s algorithm.

### 2.6 Monitoring of Binding Pocket Dynamics

First, the detection of binding sites on OSM-OSMRβ-gp130 was performed using the MDpocket [42] based on 50 frames extracted from the simulation trajectory. During calculation, two parameters of isovalue thresholds for density and frequency fields were set to 2 and 0.35 [42], respectively. Then, the flexibility of the drug binding pocket on OSM-OSMRβ-gp130 during the entire MD simulation was analyzed by POVME [43] and VMD [44]. For volume calculation, the binding pocket was defined by using the sites detected in the MDpocket analysis. POVME analyzed the whole frames from the perspective of the defined binding pocket and identified unique conformations of the target protein.

## 3 RESULTS AND DISCUSSION

### 3.1 Model of OSM-OSMRβ-gp130 Complex

In this work, the OSM-OSMRβ-gp130 model was constructed based on the crystal structures of LIF in complex with LIFR (PDB ID: 2Q7N) and gp130 (PDB ID: 1PVH) [24]. The workflow contains three steps (Fig. **2B**): (i) generating the LIFR-gp130 structure by aligning LIF-LIFR onto LIF-gp130 with LIF as a reference, (ii) constructing the OSMR-gp130 complex by aligning the full-length structures of OSMRβ and gp130 onto the LIFR and gp130, respectively, and (iii) the OSM-OSMRβ-gp130 model was finally obtained by aligning OSM onto the OSMRβ-gp130 structure using LIF as a reference.

Several complex structures of IL-6 family cytokines (*i.e.*, LIF, IL-27, and IL-6) bound to corresponding receptor(s) were determined [24, 45, 46]. Sequence analysis revealed that OSM was most similar to LIF, with a sequence identity of 24.68% between the two [17]. Additionally, OSM and LIF were most close in structure and function among the IL-6 family [4]. Therefore, the structures of the LIF-bound complex were used as templates. The result validated that the overall architecture of the OSM-OSMRβ-gp130 model was structurally similar to that of the latest reported LIF signaling complex in ternary form (Fig. **S1**) [47]. In our model, OSM formed a 1:1:1 ternary complex with OSMRβ and gp130, in which the BC loop and binding site II of OSM interacted with the OSMRβ (D2 and D3) and gp130 (D2 and D3), respectively (Fig. **2B**), which was consistent with the site mutagenesis studies [4].

**Fig. (3):**
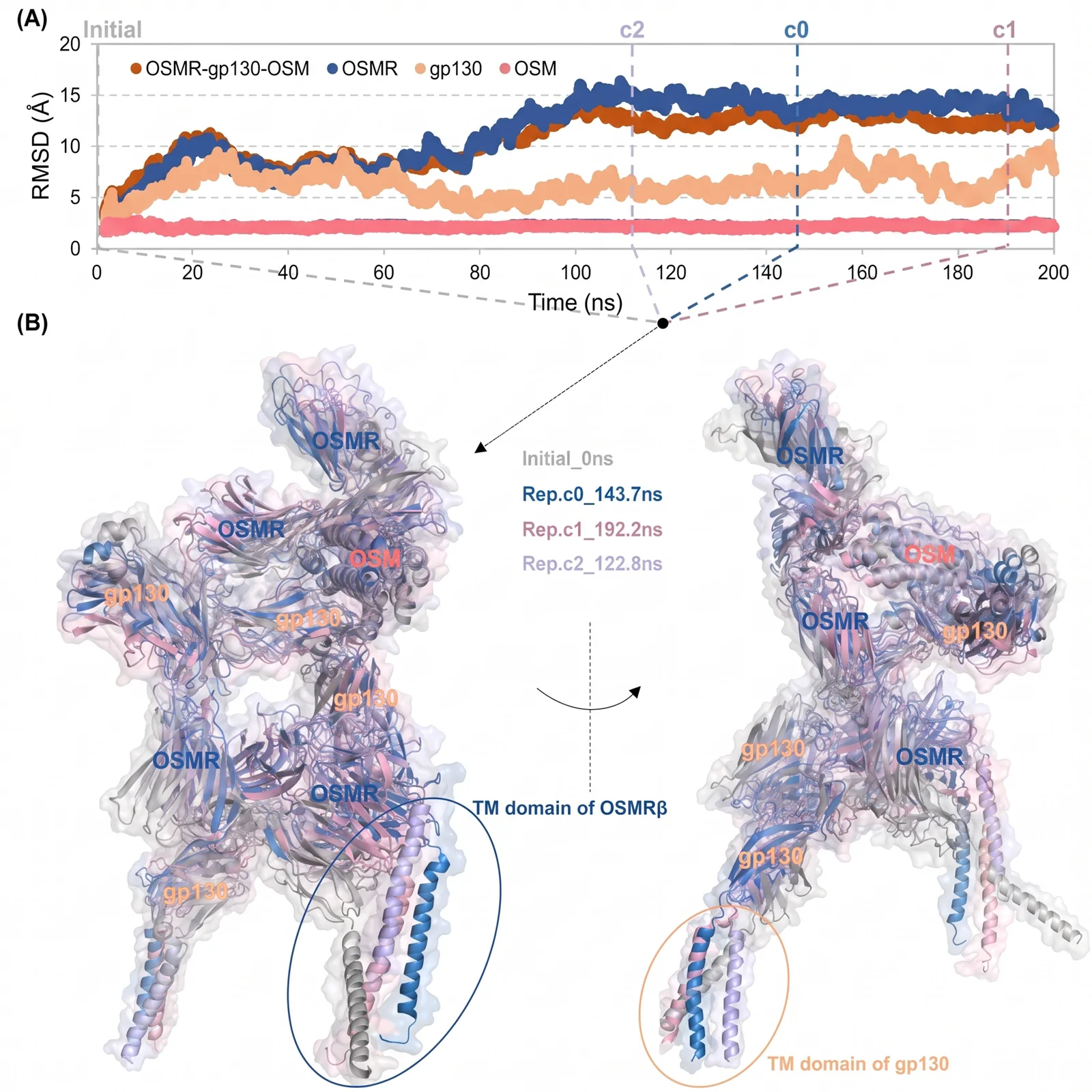
Conformational dynamics of the OSM-OSMRβ-gp130 complex. (**A**) Root-mean-square deviation (RMSD) of the protein during the 200 ns molecular dynamics (MD) simulation. (**B**) Structural alignment of the three representative conformations (Rep.c0, Rep.c1, and Rep.c2).

To further optimize the model, the 3D structure was submitted to all-atom MD simulations in an explicit solvent environment consisting of TIP3P waters and POPC lipids (~ 1.12 million atoms) [48]. The Cα RMSD values as a function of time demonstrated that the model reached equilibrium around 100 ns (Fig. **3A**). Meanwhile, the monitored RMSDs of OSM, OSMR, and gp130 indicated that the OSMRβ mainly contributed to the large fluctuations (>10 Å) of the OSM-OSMRβ-gp130 model during two simulation stages (0-20 ns and 80-100 ns). Therefore, it could be drawn from the RMSD results that the flexibility of OSMRβ was important for protein-protein recognition during the formation of the OSM-OSMRβ-gp130 complex [49].

In addition, three representative structures (*i.e.*, Rep.c0_143.7ns, Rep.c1_192.2ns, and Rep.c2_122.8ns) of OSM-OSMRβ-gp130 were identified from the equilibrated simulation trajectory (100 ns to 200 ns) through K-means clustering analysis and were aligned to the initial conformation (Initial_0ns) (Fig. **3B**). Compared to the initial coordinates, it is clear that significant conformation rearrangements occurred for the whole OSMRβ protein in the OSM-OSMRβ-gp130 complex, which was consistent with the large RMSD values, as shown in Fig. (**3A**). Interestingly, although the three representative structures were derived from the equilibrated simulation trajectory, the TM domains of the heteromeric OSMRβ/gp130 exhibited remarkable flexibilities, implying that conformational switches in these TM domains may play a crucial role in mediating signal transduction from the extracellular to the intracellular regions [3].

### 3.2 Hot Spots Responsible for OSM and Receptor Complex Recognition

**Fig. (4):**
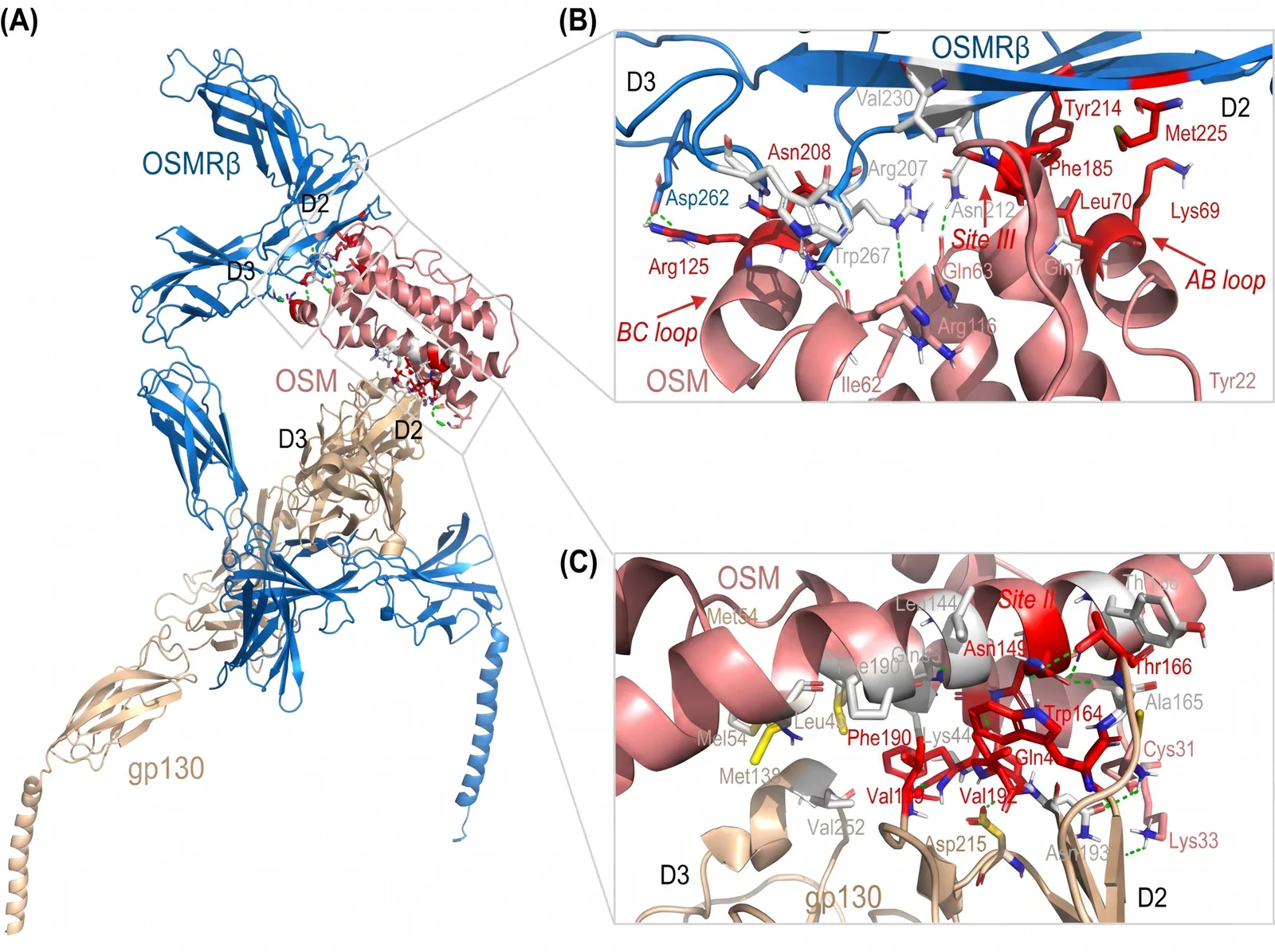
Interaction interfaces of the OSM-OSMRβ-gp130 complex. (**A**) Overview of OSM contact with OSMRβ and gp130. (**B**) The interface between OSM (AB loop, BC loop, and Site III) and D2 and D3 domains of OSMRβ. (**C**) The interface between OSM (Site II) and D2 and D3 domains of gp130. The hot spots (per-residue energy contribution higher than 2 kcal/mol) and warm spots (per-residue energy contribution between 1 kcal/mol and 2 kcal/mol) were colored in red and gray, respectively.

To quantitatively identify the hot spots residues located at the interaction interfaces that are essential for the recognition of OSM and two receptors (OSMRβ and gp130), MM/GBSA binding free energy decomposition analysis [38] was performed on the equilibrated simulation trajectory of OSM-OSMRβ-gp130 complex. As a result, 8, 3, and 5 hot spots (per-residue energy contribution higher than 2 kcal/mol) were identified on OSM, OSMRβ, and gp130 (Table **S1**), respectively. Those hot spots were further mapped on the interaction interfaces of the complex structure (Fig. **4A**). In addition, warm spots (per-residue energy contribution between 1 kcal/mol and 2 kcal/mol) were also identified and mapped in the protein-protein interaction interfaces (Fig. **4B**). As shown in Fig. (**4C**), the key residues (*i.e.*, hot spots or warm spots) that participated in interactions between OSM and OSMRβ and gp130 were located at AB loop, BC loop, Site II, and Site III of OSM, D2 and D3 domains of OSMRβ, and D2 and D3 domains of gp130. Remarkably, those interfaces were verified by the previously reported biochemical experiment results [1, 4].

### 3.3 Conformational Changes on Receptors Induced by OSM Mediate Signal Transduction

**Fig. (5):**
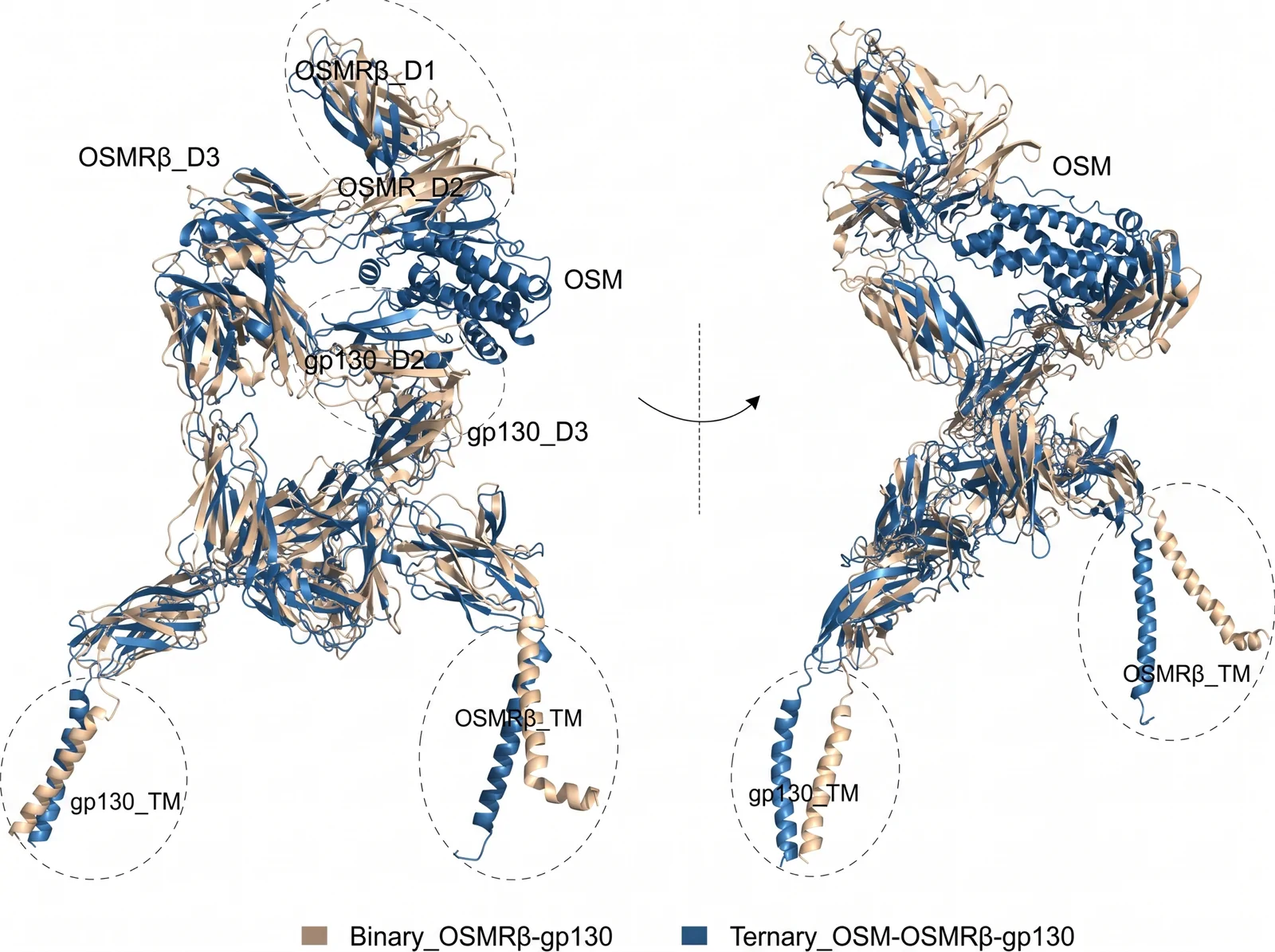
Conformational changes on OSMRβ and gp130 receptors induced by OSM. Compared with the binary structure of OSMRβ-gp130 (wheat), there are 4 main large conformational changes (D2, D3, and TM on OSMRβ, and D2, D3 and TM on gp130) on OSM-OSMRβ-gp130 complex (blue).

The structural behaviors of the receptor complex ternary models (Figs. **3A**, **3B**) suggested that upon OSM binding, specific conformational changes occurred on the receptors of OSMRβ and gp130. To deeply understand the active conformations of receptors induced by OSM, the OSMRβ-gp130 receptor complex without OSM binding (*i.e.*, inactive conformation) was constructed with its structure optimized by MD simulation using the same setups in the ternary OSM-OSMRβ-gp130 model. Compared with the representative structure of the binary OSMRβ-gp130 model without OSM binding, there were 4 main large conformational changes (D2, D3, and TM on OSMRβ, and D2, D3, and TM on gp130) on OSM-OSMRβ-gp130 complex (Fig. **5**). For the D2 and D3, large conformational changes occurred because OSM mainly interacted with the D2 and D3 interface of the two receptors when the ternary complex was formed. The structural rearrangements of the two receptors' TM domain may be induced by the long-range communications originating from the ligand-receptor interface. Research has shown that the C terminal of the TM domain of two receptors interacts with the intracellular signal proteins, such as JAKs and MAPKs [3]. Thus, it can be proposed that the structural rearrangements of the heterodimer receptor complex by OSM binding mediate the signal transduction [4, 49].

**Fig. (6):**
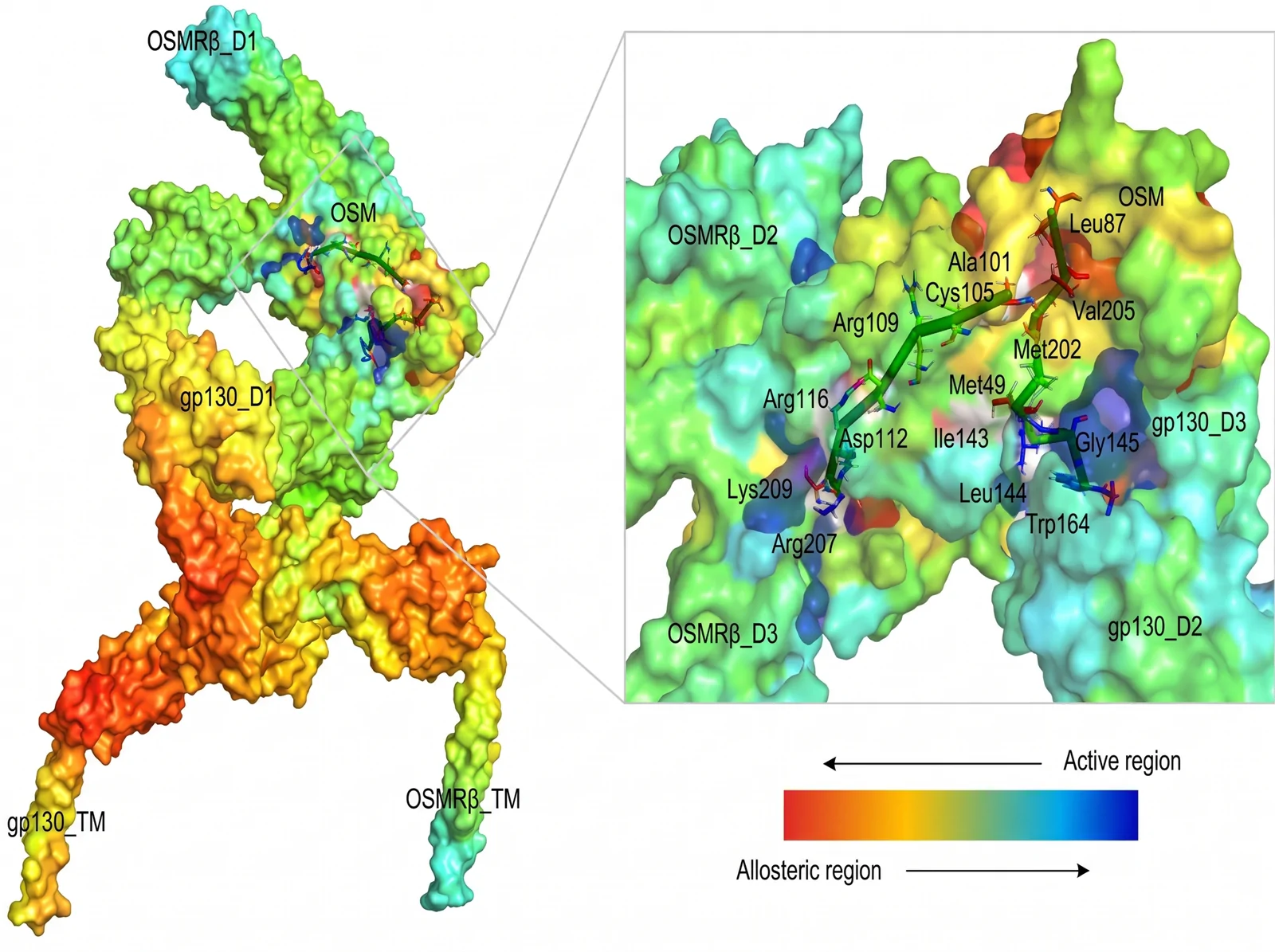
Allosteric pathways on the OSM-OSMRβ-gp130 calculated by Ohm. The two allosteric pathways in which OSM binding propagates *via* signal transduction from the OSM to OSMRβ and gp130 are shown as green cylinders. The key residues, along the pathways, are represented as sticks. The proteins are shown as surface representations and colored by ACI values. Resides with high, moderate, and low ACI values are colored yellow, green, and light blue, respectively. Residues at the allosteric region and active region of the complex are colored red and blue, respectively.

To further investigate the effects of OSM binding propagated *via* inter-residue interactions between the ligand and receptors [50], the allosteric pathways underlying protein communication were computed based on the representative snapshot of OSM-OSMRβ-gp130 (Rep.c0_143.7ns) extracted from the equilibrated simulation trajectory. As shown in Fig. (**6**), two main allosteric pathways contributed to signal transduction from the OSM’s allosteric region (red) to the active region of OSMRβ and gp130 were identified. There were 7 (Ala101-Cys105-Arg109-Asp112-Arg116 from OSM, and Arg207-Lys209 from OSMRβ) and 8 (Leu87- Val205-Met202-Met49-Ile143-Gly145-Leu144 from OSM, and Trp164 from gp130) critical residues in the dominant allosteric pathways of OSM-OSMRβ and OSM-gp130, respectively. Here, the static 3D structure was used for allosteric pathway analysis. However, MD simulation showed that the structure was coupled to dynamics (Fig. **3**), and the allostery was a manifestation of the coupling. Therefore, the red region on OSM (*e.g.*, Leu87, Ala101, Ile143, Met202, and Val205) appeared to be sensitively coupled to the rest of the complex (Fig. **6**).

### 3.4 Binding Sites on OSM-OSMRβ-gp130 Complex for Small Molecular Drug Design

Since the regulation of OSM signaling is a promising strategy for treating inflammatory diseases [51], it is interesting to explore the druggable pockets on OSM, OSMRβ, and gp130 for structure-based small molecular drug design. Herein, binding site detection was performed using the MDpocket [42], which could identify and characterize protein binding sites and pockets by analyzing the three-dimensional coordinates of protein structures in the MD simulation trajectory [52]. To efficiently identify potential binding sites, the simulation trajectory was uniformly sampled into 50 frames. Two potential druggable pockets (*i.e.*, site 1 and site 2) were identified upon overlaying by adjusting the isovalue thresholds for density and frequency fields to 2 and 0.35, respectively. As shown in Fig. (**7**), site 1 was situated within the open cavity of OSM, whereas site 2 was located at the hinge region connecting gp130 and OSM.

**Fig. (8):**
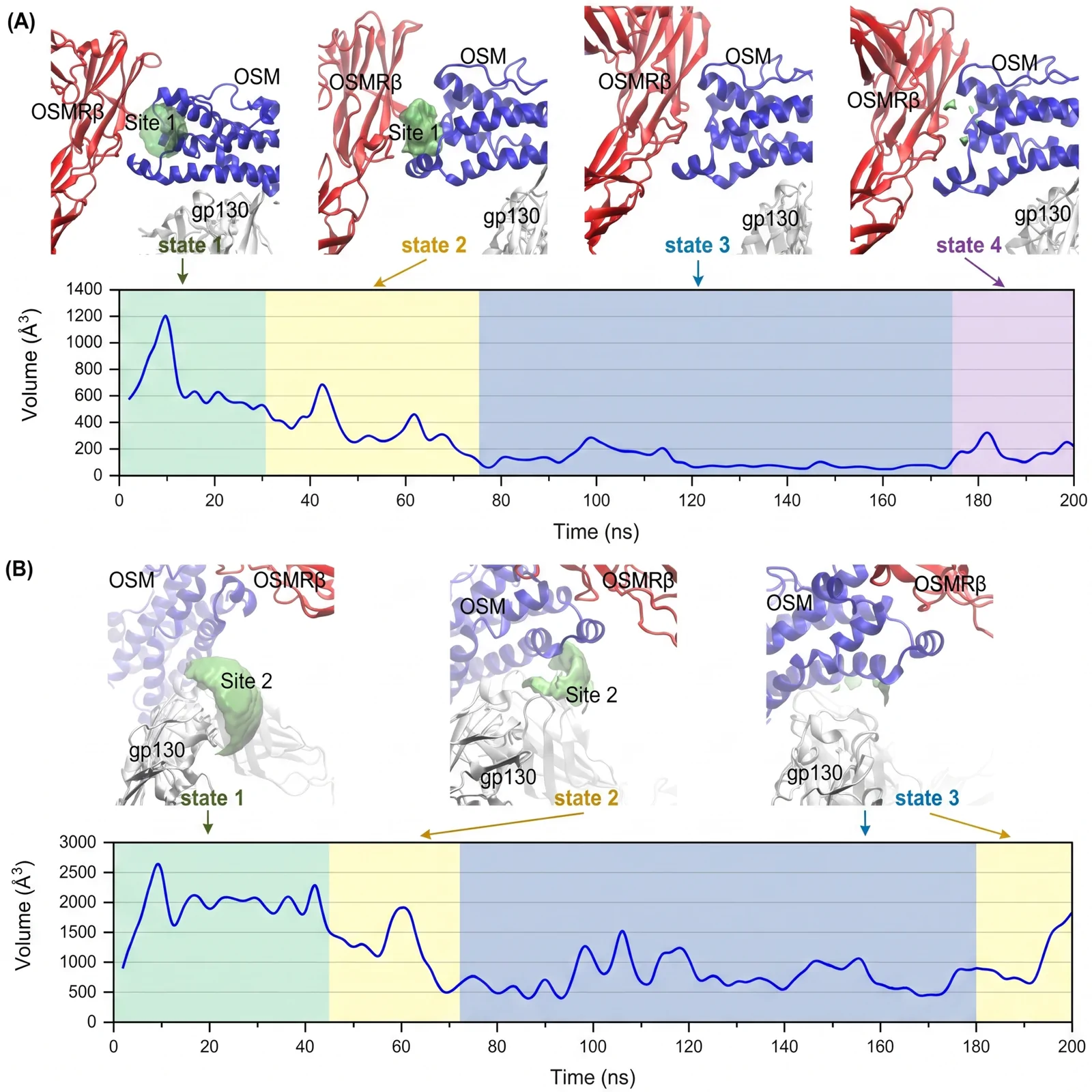
The volume changes and clustering of the two pockets calculated by POVME. (**A**) The volume changes and classification stages of site 1. (**B**) The volume changes and classification stages of site 2. The green isosurface indicates regions relatively more open compared to the average volume. The black isosurface indicates regions relatively more closed compared to the average volume.

Based on site 1 and site 2 detected by MDpocket, the flexibility of the pockets (*i.e.*, volume changes) during the entire MD trajectory was further analyzed by POVME [43] and VMD [44]. According to the k-means clustering algorithm, each of the pockets was classified into three or four clusters, representing distinct stages along the simulation (Fig. **8**). Monitoring the volume of site 1 indicated that the druggable pocket disappeared when OSM tightly bound to OSMRβ (Fig. **8A**), suggesting that the design of small molecules occupying the pocket may block the recognition between OSM and OSMRβ. Notably, site 1 was consistent with the binding site of SMI-10B (a first-in-class small molecule) targeting OSM [53] identified by MD simulations [18]. Similarly to site 1, the volume of site 2 decreased continuously during the binding process of OSM with gp130 (Fig. **8B**). As a result, the design of small molecules targeting gp130 at this site could potentially inhibit the interaction between OSM and gp130 and thus obstruct the intracellular signal transduction.

It is notable that residues comprising site 1 and site 2 (Table **1**) include Phe185 on OSM and Val189 on gp130, which have been identified as hot spots that are essential for the ternary complex formation. Starting with the sites identified on OSM and gp130, one can utilize computational methods, such as structure-based virtual screening [54] and artificial intelligence-driven generative models [55], to rationally design small molecules [18] that inhibit the interaction between OSM and the heteromeric receptors OSMRβ and gp130. Compared to traditional mAbs, small molec- ules offer several advantages, including better tissue penetration, enhanced stability, and reduced manufacturing costs [13, 15]. Therefore, our model has the potential to make a significant impact on drug discovery aimed at targeting OSM signaling. One limitation of this study is the absence of independent simulations. Nevertheless, all input files, parameter files, topology files, and trajectory analysis scripts used in this work are provided as supplementary data. This will facilitate further research on the OSM-OSMRβ-gp130 ternary complex.

## CONCLUSION

In this study, for the first time, we report a structural model of the OSM-OSMRβ-gp130 ternary complex using deep learning-based AlphaFold2 protein structure prediction and all-atom MD simulation, guided by the structural information from the experimental homologues of LIF in complex with LIFR and gp130 as templates. A comprehensive analysis revealed that OSM binds to OSM-gp130 by an induced-fit mechanism and triggers the signaling through an allosteric communication network upon ternary complex formation. These findings provide valuable insights into the molecular basis of OSM signaling and identify druggable sites for the rational design of novel therapeutics aimed at modulating OSM signaling and inhibiting intracellular signal transduction.

## Figures and Tables

**Fig. (7):**
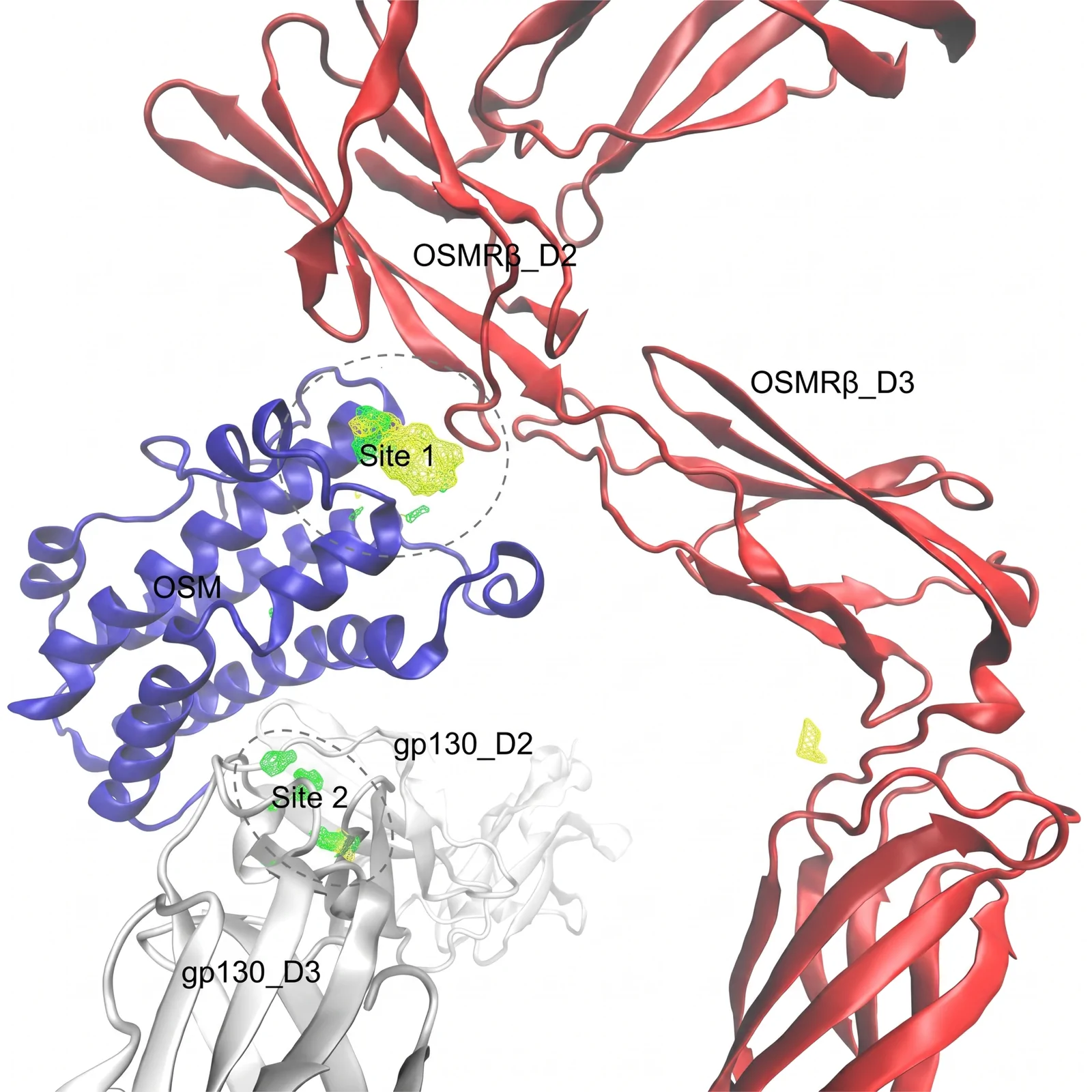
Two binding sites on the OSM-OSMRβ-gp130 complex revealed by MDpocket analysis. The density isosurfaces are depicted by green wireframes, while the frequency isosurfaces are represented by yellow wireframes.

**Table 1:** List of residues that comprise site 1 and site 2 in the OSM-OSMRβ-gp130 model.

**Proteins**	**Stages**	**Volume**	**Residues**
OSM	1	724 Å3	Ser53, Tyr59, Ile62, Gly64, Arg116, Leu117, Pro118, **Phe185** *a*, Leu189, Gly191, Cys192
	2	335 Å3	Ile62, Gln63, Gly64, Arg116, Leu117, Pro118, **Phe185** *a*, Lys188
gp130	1	2154 Å3	Asn137, Glu138, Gly139, Lys140, Tyr186, Ser187, Thr188, **Val189** *a,* Val220, Lys221, Pro222, Asn223, Pro247, Ser248, Ile249
	2	775 Å3	Glu138, Gly139, Lys140, Lys141, Tyr186, Ser187, Thr188, **Val189** *a*

**Note:** *a* Residues identified as hot spots that are essential for the OSM-OSMRβ-gp130 complex formation.

## Data Availability

The input files, parameter files, topology files, and trajectory analysis scripts used to support the findings of this study are provided as Supporting Information. The MD trajectory in this work is available in Zenodo at https://zenodo.org/uploads/13621875

## LIST OF ABBREVIATIONS

OSM: Oncostatin M
OSMRβ: OSM Receptor Beta
gp130: Glycoprotein 130
IBD: Inflammatory Bowel Disease
RA: Rheumatoid Arthritis
MD: Molecular Dynamics
IL-6: Interleukin 6
CNTF: Ciliary Neurotrophic Factor
LIF: Leukemia Inhibitory Factor
CT-1: Cardiotrophin 1
JAK: Janus Kinase
STAT: Signal Transducer and Activator of Transcription
MAPK: Mitogen-activated Protein Kinase
mAbs: Monoclonal Antibodies
PPIs: Protein-protein Interactions
pLDDT: Predicted Local-distance Difference Test
PME: Particle-Mesh Ewald
RMSD: Root-mean-square Deviation
ACI: Allosteric Coupling Intensity
