# Supplementary material for: Structural Model of the Oncostatin M (OSM)-OSMRβ-gp130 Ternary Complex Reveals Pathways of Allosteric Communication in OSM Signaling
Source: Curr Med Chem. 2025 Jul 1;33(21):4380–94. doi: 10.2174/0109298673362742250508113436 (PMC13555842; doi:10.2174/0109298673362742250508113436)
Supplement: Supplementary file 1 [file CMC-33-21-4380_SD1.pdf]

Supplementary Material

Structural Model of the Oncostatin M (OSM)-OSMRβ-gp130 Ternary Complex Reveals Pathways of Allosteric Communication in OSM Signaling

Qingqing Du[1], Ding Luo[2], Weiwei Xue[2,*] and Yan Qian[1,*]

[1]Department of Pharmacy, The Second Affiliated Hospital of Chongqing Medical University, Chongqing, 400010, China; [2]Chongqing Key Laboratory of Natural Product Synthesis and Drug Research, School of Pharmaceutical Sciences, Chongqing University, Chongqing, 401331, China

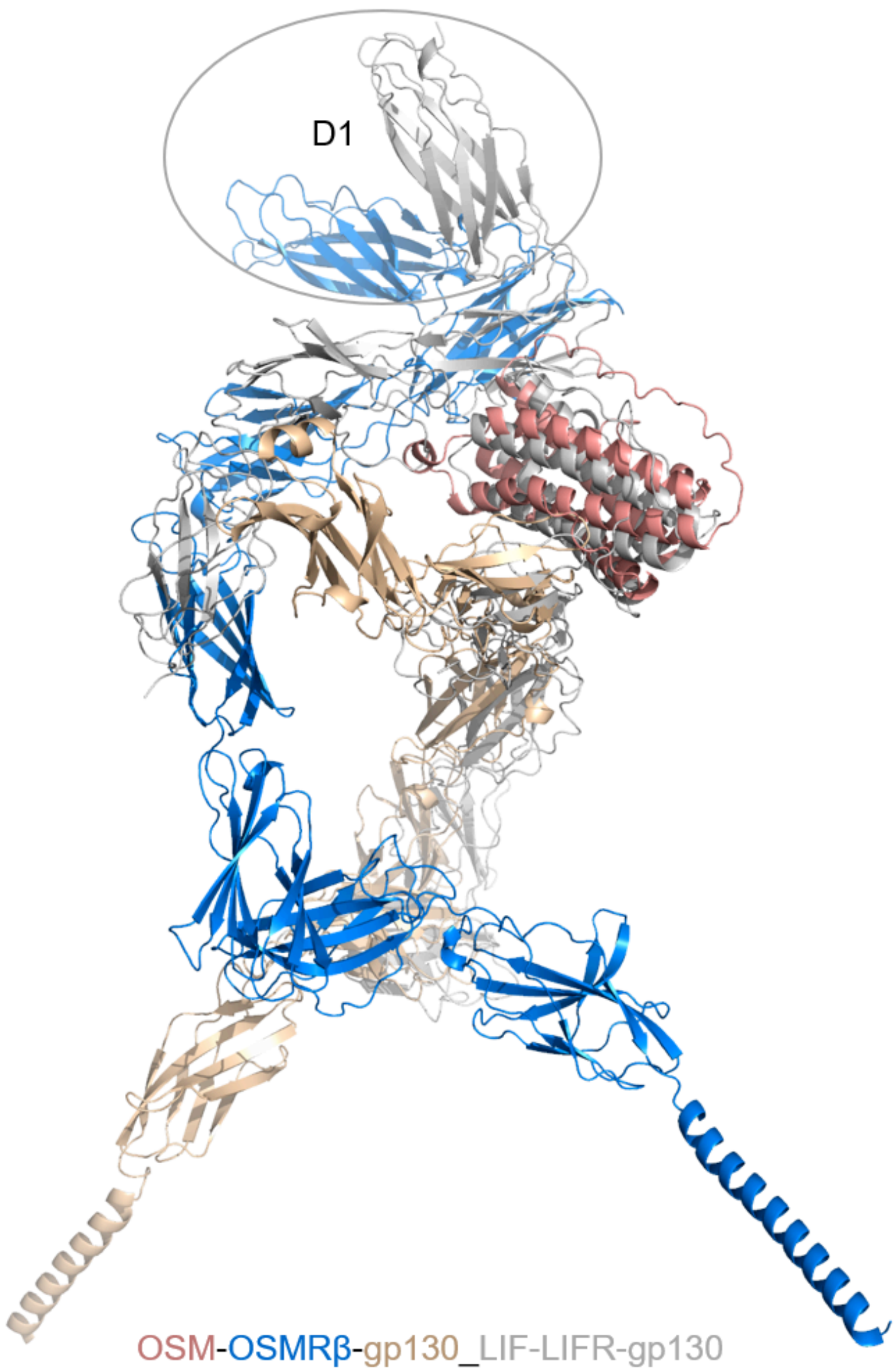

Fig. (S1). Overall structural alignment between the OSM-OSMRβ-gp130 model and the latest reported LIF signaling complex in ternary.

Table S1. Energy contributions (kcal/mol) of the hot spots and warm spots identified on OSM, OSMRβ, and gp130, respectively.

Proteins	OSM		OSMRβ		gp130	
Residues	Number	Energy	Number	Energy	Number	Energy
Hot Spots	41	-4.74	208	-2.29	164	-3.76
	69	-4.3	214	-2.19	166	-4.25
	70	-2.18	226	-2.98	189	-2.88
	125	-3.32			191	-6.09
	126	-2.69			192	-3.83
	148	-3.45				
	149	-2.2				
	185	-5.34				
Warm Spots	44	-1.49	207	-1.4	165	-1.96
	45	-1.45	212	-1.53	193	-1.32
	48	-1.98	230	-1.47	252	-1.17
	54	-1.69	267	-1.18		
	67	-1.49				
	138	-1.26				
	141	-1.31				
	144	-1.8				
	145	-1.23				
	151	-1.23				
